# Association Between SNPs of Long Non-coding RNA *HOTAIR* and Risk of Different Cancers

**DOI:** 10.3389/fgene.2019.00113

**Published:** 2019-02-28

**Authors:** Mohammadreza Hajjari, Saghar Rahnama

**Affiliations:** Department of Biology, Faculty of Science, Shahid Chamran University of Ahvaz, Ahvaz, Iran

**Keywords:** HOTAIR, lncRNA, cancer, SNP, polymorphism

Long non-coding RNAs (LncRNAs) are RNAs with more than 200 nucleotides and are mostly transcribed by RNA polymerase II from different regions across the genome. They are currently known as key regulators of cellular function through different mechanisms such as epigenetic regulation, miRNA sponging, and modulating of proteins and enzyme cofactors (Kurokawa, 2011; Nie et al., 2012; Flynn and Chang, 2014; Birgani et al., 2017; Marchese et al., 2017). By this way, they are implicated in development pathways (Amaral and Mattick, 2008). Different lncRNAs such as *HOTAIR* can play their important roles by changing the chromatin states of the genome (Mercer and Mattick, 2013). Rinn et al. introduced this RNA as a spliced and polyadenylated RNA with 2,158 nucleotides (Hajjari et al., 2013). *HOTAIR*, as one of the featured lncRNAs, is located between *HOXC11* and *HOXC12* on chromosome 12q13.3. *HOTAIR* forms stem-loop structures which bind to histone modification complexes lysine-specific demethylase 1 (LSD1) and Polycomb Repressive Complex2 (PRC2) in order to recruit them on specific target genes. This RNA interacts with Polycomb repressive Complex2 (PRC2) and has a lot of targets such as *HOXD*. By this way, PRC2 can repress the desired genes leading into increased growth, proliferation, survival, metastasis, invasion, and drug resistance in some cancer cells (Rinn et al., 2007; He et al., 2011; Davidovich et al., 2013; Hajjari et al., 2014; Martens-Uzunova et al., 2014; Zhao et al., 2014). So, different studies have indicated the dysregulation of *HOTAIR* in different types of cancers in recent years (Gupta et al., 2010; Kogo et al., 2011; Yang et al., 2011; Niinuma et al., 2012; Hajjari et al., 2013; Kim et al., 2013; Li et al., 2013).

In recent studies, there are some reports indicating the role of *HOTAIR* SNPs which make it a significant cancer susceptibility locus and provide high risk for some cancers (Qi et al., 2016), like breast (Bayram et al., 2015, 2016; Yan et al., 2015), gastric (Pan et al., 2016; Tian et al., 2016), cervical (Guo et al., 2016; Qiu et al., 2016), papillary thyroid carcinoma (Zhu et al., 2016), osteosarcoma (Zhou et al., 2016), prostate (Taheri et al., 2017), ovarian (Wu et al., 2016; Qiu et al., 2017), and colorectal cancers (Xue et al., 2014). This is an interesting point because these SNPs may have effect on gene expression, function, and regulators of epigenome (Hajjari and Rahnama, 2017). Therefore, we think that more studies on these SNPs can reveal the potential of these SNPs for considering them as markers of progression and diagnosis of different cancers.

Figure 1 shows the locations of these SNPs within *HOTAIR* gene. Herein, we present different SNPs to highlight their potential for further studies.

**Figure 1:**
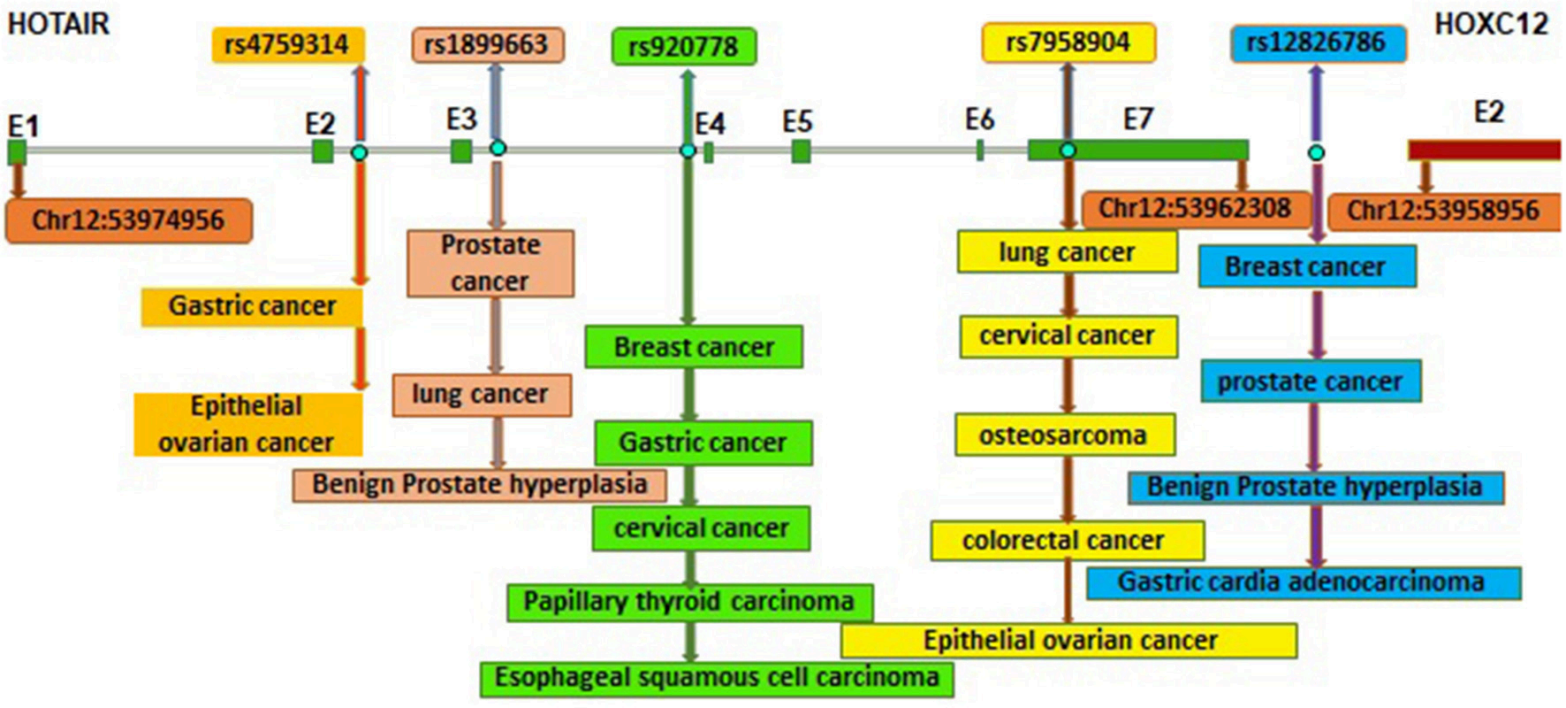
Locations of different SNPs within *HOTAIR* gene and their association with different types of cancer (E: Exon, exons of HOTAIR, and HOXC12 are shown by green and red boxes). Genomic positions are based on the UCSC Genome browser on Human Dec. 2013 (GRCh38/hg38) assembly.

There are some reports indicating the association between *HOTAIR* rs12826786 SNP which is located between *HOTAIR* and *HOXC12*. The increased risk for some cancers such as breast (BC) (Bayram et al., 2016), gastric adenocarcinoma (GCA) (Guo et al., 2015), prostate cancer (PC), and benign prostate hyperplasia (BPH) (Taheri et al., 2017) has been reported. For instance, women who are carriers of this polymorphism, have an increased risk of BC in both codominant and recessive inheritance models (Bayram et al., 2016). With regard to the location of this SNP, it seems that this SNP has effect on the regulation of *HOTAIR* gene in the cell. So, the analysis of *HOTAIR* dysregulation and its correlation with this SNP can be proposed in different types of cancers in different population.

rs920778 is another polymorphism which is located in the intronic enhancer of *HOTAIR* gene. TT genotype of this SNP has been found to affect the gene expression and make the risk for various cancers (Bayram et al., 2015) such as gastric (Pan et al., 2016), esophageal squamous cell carcinoma (Zhang et al., 2014), cervical (Qiu et al., 2016), and papillary thyroid carcinoma (Zhu et al., 2016). In addition, CC genotype of this SNP might be a cause of breast cancer in both codominant and recessive inheritance genetic models (Bayram et al., 2015).

There are some studies reporting the association between the dysregulation of *HOTAIR* and rs920778. *HOTAIR* up-regulation has been suggested as a result of rs920778 in gastric cancer (Xu et al., 2013; Pan et al., 2016). Also, the aberrant expression of *HOTAIR* in esophageal squamous cell carcinoma seems to be the result of a specific allele of rs920778 (Gupta et al., 2010; Zhang et al., 2014; Dai et al., 2017). Furthermore, there is higher expression of *HOTAIR* in female papillary thyroid carcinoma tissues because of a specific genetic polymorphism of this gene (Zhu et al., 2016).

Another SNP annotated as rs4759314 is also located in a promoter region in one of the introns of *HOTAIR*. It is of noted that AG/GG genotypes of the rs4759314 were associated with gastric cancer risk. The expression effects of heterozygotes individuals with G allele were more than homozygotes in the patients in co-dominant models (Du et al., 2015). However, in a controversial report, the *HOTAIR* gene expression found to be higher in ovarian cancer patients with AG/AA genotypes of rs4759314 (Wu et al., 2016).

Another SNP located in the intronic region of *HOTAIR* is rs1899663. Due to its location in a putative regulatory element, it seems that this SNP can affect gene expression and regulation. There are some association between *HOTAIR* rs1899663 T allele and BPH (Benign prostate hyperplasia) patients. Also, The rs1899663 is associated with prostate cancer risk in co-dominant, dominant and recessive inheritance models. Researchers have reported that this SNP changes the affinity for binding of PAX-4, SPZ1, and ZFP281 transcription factors which can alter the *HOTAIR* gene expression level (Taheri et al., 2017).

Among the SNPs in *HOTAIR* gene, one named “rs7958904” is an exonic polymorphism. So, it seems that *HOTAIR* rs7958904 polymorphism can affect the secondary structure of *HOTAIR*.

It is of noted that CC genotypes of *HOTAIR* rs7958904 has been reported to be associated with decreased osteosarcoma (Zhou et al., 2016), EOC (Wu et al., 2016), and colorectal cancers risk (Xue et al., 2014). In an study on osteosarcoma patients classified by age, gender, and tumor locations, it was shown that CC genotypes of the *HOTAIR* rs7958904 can reduce osteosarcoma risk as well as *HOTAIR* expression level (Zhou et al., 2016). However, cervical cancer patients with CC genotypes of this SNP had higher *HOTAIR* expression (Jin et al., 2017). Furthermore, with regard to the up-regulation of *HOTAIR* in lung cancer (Jiang et al., 2017) the SNP has been reported as a region to be associated with chemotherapy response in lung cancer patients through effect on *HOTAIR* function or expression (Xue et al., 2014; Gong et al., 2016).

*HOTAIR* have abnormal expression in the different human cancers. Different studies have revealed the cellular and molecular mechanisms in which *HOTAIR* is involved (Hajjari and Salavaty, 2015; Gong et al., 2016). Recently, some studies indicating the potential role of SNPs of *HOTAIR* in cancer susceptibility have been published. However, these studies are mostly derived from Asian population. Also, there are some controversial results on this field of study. With regard to the importance of *HOTAIR* regulation and function, more experiments on different populations, and ethnics are expected to reveal the importance of *HOTAIR* polymorphisms. Other polymorphisms in *HOTAIR* gene such Indel and CNV may be considered in future. However, the association between these SNPs and regulation/structure of *HOTAIR* has to be checked in various cancers. Also, we believe that whole genome sequencing projects can help to find the relation between the SNPs of this RNA with other SNPs in different cancers in future.

## Author Contributions

MH designed and wrote the manuscript. SR worked on gathering the data and wrote the manuscript.

### Conflict of Interest Statement

The authors declare that the research was conducted in the absence of any commercial or financial relationships that could be construed as a potential conflict of interest.
